# Natural environment satisfaction moderates the protective effect of legal cognition on adolescent aggression

**DOI:** 10.3389/fpsyg.2025.1668723

**Published:** 2025-10-02

**Authors:** Lu Fan, Yan Chen, Xuee Zou, Shuhui Xu

**Affiliations:** ^1^Department of Psychology, Wenzhou University , Wenzhou, China; ^2^College of International Education, Wenzhou University, Wenzhou, China

**Keywords:** legal cognition, aggressive behavior, natural environment satisfaction, legal education, protective factors for aggression

## Abstract

Adolescent aggression poses a threat to community wellbeing and sustainable crime prevention. Grounded in situational crime prevention and social control theories, this study examined whether legal cognition (understanding of legal norms and rights) predicts lower aggression and whether natural environment satisfaction moderates this effect. In spring 2024, 518 adolescents (12–22 years) in Zhejiang Province completed validated scales of legal cognition, aggression, and environmental satisfaction. Hierarchical regression and PROCESS Model 1 analyses showed that legal cognition negatively predicted aggression (*β* = −0.34, *p* < 0.001) and that environmental satisfaction also inversely related to aggression (*β* = −0.30, *p* < 0.001). Critically, the legal cognition × environment interaction explained an additional 4.5% of aggression variance (Δ*R*^2^ = 0.045, *p* = 0.002), with the buffering effect of legal cognition being strongest at high environmental satisfaction. These results suggest that combining legal-norm education with enhancements to natural settings (e.g., increased green space, reduced pollution) may offer a scalable strategy for adolescent violence prevention.

## 1 Introduction

Aggression refers to an individual’s tendency to engage in behaviors intended to harm others (Anderson and Bushman, 2002). Aggressive behavior is a prevalent issue among adolescents and represents a significant manifestation of social maladjustment (Kamper and Ostrov, 2013). Previous research has primarily focused on the influence of social environmental factors on aggression, such as the role of various social relationships in shaping aggressive behaviors (Fan et al., 2024; Hu et al., 2025). However, relatively little attention has been given to the potential role of natural environmental factors in this process.

Given that adolescence is a critical period for cognitive development, individuals at this stage are highly susceptible to external environmental influences, which may contribute to behavioral problems. Research has shown that the incidence of aggressive behavior is particularly high during this developmental phase (Estévez et al., 2020; Zhou et al., 2025). At the same time, adolescence is also a crucial period for the development of legal cognition (Trinkner et al., 2018). Legal cognition not only affects an individual’s understanding and adherence to social norms but may also play a regulatory role in aggressive behavior. However, existing studies have rarely examined how legal cognition interacts with natural environmental factors to influence adolescent aggression.

From a crime prevention perspective, this study employs a legal cognition × natural environment satisfaction research design to investigate the interactive effects of these factors on adolescent aggressive behavior. The findings are expected to provide both theoretical insights and practical guidance for the prevention and intervention of adolescent aggression.

## 2 Literature review and hypotheses

### 2.1 Legal cognition and aggressive behavior

Legal cognition encompasses adolescents’ abstract understanding of the nature, values, and functions of law, as well as their concrete grasp of rights and obligations, exerting a profound influence on normative awareness and behavioral choices (Xu and Yan, 2022). When adolescents perceive law as a social contract that upholds public will and social order and endorse its core values of fairness, justice, freedom, and order, their willingness to comply with rules and sense of social responsibility increase markedly, making them more likely to resolve conflicts through rational, lawful means (Espy, 2004; Hirschi, 2002; Xu and Yan, 2022). Moreover, a clear understanding of one’s legal rights—and the boundaries within which they may be exercised—promotes respect for others’ entitlements and suppresses impulses to use aggression in defense of self-interest; internalizing legal obligations further strengthens norm adherence and reduces the likelihood of hostile or unlawful acts (Hirschi, 2002; Xu et al., 2024).

Adolescent aggression can be categorized as emotionally driven reactive aggression, goal-directed proactive aggression, and displaced aggression—where frustration or provocation leads an individual to redirect anger toward an innocent target. Displaced aggression typically rises during early adolescence and peaks in middle school (Anderson and Bushman, 2002; Denson et al., 2006; Garcia-Sancho et al., 2016; Raine et al., 2006; Rajchert et al., 2022). These forms of aggression are linked to academic decline, substance misuse, and later violent offending, and constitute a major public-health burden (Connor et al., 2004; Farrington, 1994; Loeber and Dishion, 1983; Rowell et al., 2009; Shepard et al., 2016).

Extensive empirical evidence shows that higher levels of legal cognition are associated with lower rates of aggressive and delinquent behavior, whereas negative or cynical attitudes toward law correlate with increased proactive and displaced aggression and criminal risk (Kaiser and Reisig, 2019; Nivette et al., 2019; Sampson and Bartusch, 1998). Although prior research has thoroughly examined risk factors such as parental conflict, relative deprivation, moral disengagement, and abuse experiences (Pan et al., 2024; Zhou et al., 2025; Zou et al., 2024), the protective role of legal cognition remains underexplored. Grounded in Social Control Theory—which posits that strong bonds to legal and moral norms inhibit deviance (Hirschi, 2002)—this study aims to systematically examine the predictive effects of legal cognition on various forms of aggressive behavior to inform law-education interventions and violence-prevention strategies. Therefore, we propose:

*H1:* Legal cognition negatively predicts aggressive behavior.

### 2.2 The moderating effect of natural environment satisfaction

Human behavior is fundamentally guided by hedonic motivation, whereby individuals seek pleasure and avoid discomfort (Aaker and Lee, 2001). This principle extends to perceptions of the natural environment: in the present study, natural environment satisfaction denotes an individual’s subjective appraisal of their everyday surroundings—encompassing green-space availability, wastewater treatment efficacy, air quality, and general ecological management. While extant research has largely emphasized social contexts, our focus shifts to how the natural milieu itself shapes psychological and behavioral outcomes.

Several theoretical models illuminate these effects. The Biophilia Hypothesis (Wilson, 1984), Stress Reduction Theory (Ulrich et al., 1991), and Attention Restoration Theory collectively posit that exposure to nature reduces stress (Kaplan and Kaplan, 1989), restores cognitive resources, and enhances mental functioning (Han, 2001). Empirical work corroborates these claims: greater residential greenery correlates with lower rates of mental-health disorders (Gascon et al., 2018), whereas chronic exposure to air pollution or traffic noise elevates psychological distress (Kioumourtzoglou et al., 2017; Zijlema et al., 2016). From a developmental perspective, Ecological Systems Theory highlights the environment’s pivotal role in adolescent growth (Bronfenbrenner and Evans, 2000), for instance, Liao et al. (2020) found that neighborhood greenness inversely predicts youth aggression, and a large twin study in Southern California reported that higher residential greenery is linked to reduced aggressive behavior (Younan et al., 2016).

Integrated within the General Aggression Model and the Social Information Processing framework (Anderson and Bushman, 2002; Crick and Dodge, 1994), external environments are understood to influence aggression by altering cognitive appraisal, cue interpretation, and response selection. Accordingly, individuals’ satisfaction with their natural surroundings may strengthen—or weaken—the regulatory impact of legal cognition on aggressive tendencies. Grounded in this theoretical and empirical foundation, we hypothesize:

*H2:* Natural environment satisfaction moderates the relationship between legal cognition and aggressive behavior.

## 3 Methods

### 3.1 Participants

In spring 2024, we employed a convenience random sampling method at a middle school and a university in Zhejiang Province and distributed 523 questionnaires. Five were excluded because they had over 10% missing responses, exhibited numerous identical answers, or were completed in an abnormally short time (“Abnormally short time” was defined as completing the questionnaire in less than 5 min, which is substantially shorter than the expected 20–25 min completion time based on pilot testing data, suggesting potential careless or inattentive responding). This left 518 valid questionnaires (99.04% valid response rate). The final sample comprised 173 males and 345 females, aged 12–24 years (*M* = 18.23, *SD* = 3.38).

### 3.2 Measures

The Adolescent Aggression Questionnaire, adapted from the Aggression Questionnaire developed by Buss and Perry (1992) and revised for Chinese populations by Liu et al. (2009), was used to assess aggressive behavior. The questionnaire consists of four dimensions: physical aggression (e.g., “I sometimes have uncontrollable urges to hit someone.”), anger (e.g., “When I experience frustration and failure, I express my anger.”), hostility (e.g., “Sometimes I feel like I have been treated unfairly in life.”), and displaced aggression (e.g., “I have broken things out of frustration.”). The scale includes 21 items, rated on a 5-point Likert scale ranging from 1 = “strongly disagree” to 5 = “strongly agree.” The revised Chinese version demonstrated strong psychometric properties, with a homogeneity reliability coefficient of 0.84 and a test–retest reliability of 0.92 (Liu et al., 2009). In the present study, Cronbach’s alpha was 0.917, indicating excellent internal consistency.

The Adolescent Legal Cognition Scale, adapted from Xu (2019), consists of 29 items rated on a 5-point Likert scale (1 = “strongly disagree” to 5 = “strongly agree”), with higher scores indicating greater legal cognition. Example items include “I know that one cannot disclose another person’s privacy without permission” and “I believe that parents have the obligation to raise and educate their minor children.” The scale has demonstrated good psychometric properties in previous studies (CFA: CMIN/df = 1.92, GFI = 0.92, NFI = 0.90, IFI = 0.95, TLI = 0.93, CFI = 0.95, RMSEA = 0.06; internal consistency *α* = 0.97; split-half reliability = 0.77). In the present study, Cronbach’s alpha was 0.987, indicating excellent internal consistency.

The Natural Environment Satisfaction Survey was adapted from the Yunnan Province Environmental Satisfaction Survey (Liu, 2020). The scale consists of 14 items assessing environmental satisfaction, including air quality, greenery, wastewater treatment, waste disposal, sanitation facilities, ecological conditions, and pollution levels. Example items include: “I am satisfied with the air quality in my living area,” “I am satisfied with the ecological environment in my living area,” and “There are many green plants in the area where I live.” The scale is rated on a 5-point Likert scale ranging from 1 = “strongly disagree” to 5 = “strongly agree,” with higher scores indicating greater satisfaction. The revised scale demonstrated good psychometric properties, with CFA indicating *χ*^2^/df = 1.56, GFI, TLI, IFI, and CFI all above 0.90, RMSEA = 0.08, and internal consistency reliability *α* = 0.78 (Liu, 2020). In the present study, Cronbach’s alpha was 0.831, demonstrating good internal consistency.

### 3.3 Procedure and statistical analysis

The study was conducted by graduate students in psychology, who served as principal investigators. The survey was administered during students’ break periods in a group setting. Before the participants completed the questionnaire, the principal investigator read the instructions aloud, emphasizing the importance of independent responses and ensuring the anonymity and confidentiality of the data. All participants provided informed consent, with parental consent obtained for minors. After data collection, SPSS 21.0 was used for descriptive statistics and correlation analysis. Additionally, the PROCESS macro developed by Hayes (2013) was employed to test the proposed model.

## 4 Results

### 4.1 Common method bias assessment and data normality evaluation

Harman’s single-factor test indicated that 12 factors had eigenvalues >1, with the first factor explaining 31.23% of variance, below the 40% threshold, suggesting common method bias was not a major concern. Data normality (*N* = 518) was assessed via skewness and kurtosis: aggressive behavior was approximately symmetric (skewness = 0.27, kurtosis = −0.17), legal cognition showed negative skewness and leptokurtosis (skewness = −2.14, kurtosis = 4.54), and natural environment satisfaction was symmetric with slightly elevated kurtosis (skewness = 0.00, kurtosis = 3.98). Despite legal cognition exceeding conventional thresholds (Kline, 2016), parametric tests were used, with key results validated through 5,000 bootstrap resamples to ensure robustness.

### 4.2 Descriptive statistics and correlation coefficients

Correlation analysis showed that legal cognition was significantly negatively correlated with aggressive behavior and positively correlated with natural environment satisfaction. Additionally, natural environment satisfaction was significantly negatively correlated with aggressive behavior. The results are presented in Table 1.

**Table 1:** Descriptive statistics and correlation analysis of study variables (*n* = 518).

Variables	1	2	3
1. Legal cognition	1		
2. Aggressive behavior	−0.21^***^	1	
3. Natural environment satisfaction	0.34^***^	−0.12^**^	1
*M ± SD*	4.61 ± 0.67	2.15 ± 0.70	3.48 ± 0.69
*M ± SD* (*Ma*)	4.53 ± 0.74	2.20 ± 0.74	3.50 ± 0.70
*M ± SD* (*Fe*)	4.64 ± 0.64	2.21 ± 0.67	3.47 ± 0.68

***p < 0.001, **p < 0.01; All values represent Pearson correlation coefficients (two-tailed). *M*, Mean; *SD*, Standard Deviation; *Ma*, Male; *Fe*, Female.

### 4.3 Gender and parental education differences in key variables

Independent samples *t*-tests were conducted to examine gender differences in legal cognition, natural environment satisfaction, and aggressive behavior. Descriptive statistics indicated that males (*n* = 173) and females (*n* = 345) scored similarly on legal cognition (male: *M* = 4.53, *SD* = 0.74; female: *M* = 4.64, *SD* = 0.64), natural environment satisfaction (male: *M* = 3.50, *SD* = 0.70; female: *M* = 3.47, *SD* = 0.68), and aggressive behavior (male: *M* = 2.20, *SD* = 0.74; female: *M* = 2.12, *SD* = 0.69). Inferential statistics revealed a violation of homogeneity of variance for legal cognition (*F* = 8.208, *p* = 0.004); thus, Welch’s *t*-test was applied, showing no significant gender difference (*t*(304.0) = −1.69, *p* = 0.093, Cohen’s *d* = −0.17). No significant differences were found for natural environment satisfaction (*t*(516) = 0.47, *p* = 0.640, *d* = 0.04) or aggressive behavior (*t*(516) = 1.18, *p* = 0.238, *d* = 0.11). All effect sizes were negligible (*d* < 0.20), indicating no meaningful gender differences in these variables.

A two-way multivariate analysis of variance (MANOVA) was performed to assess the effects of parental education and gender on the combined dependent variables. Results showed a significant main effect of parental education (Wilks’ Λ = 0.970, *F*(12, 2709.541) = 2.648, *p* = 0.002, partial *η*^2^ = 0.010), suggesting that parental education level significantly influenced children’s psychosocial outcomes. Neither the main effect of parental gender (Wilks’ Λ = 0.999, *F*(3, 1,024) = 0.487, *p* = 0.691, partial *η*^2^ = 0.001) nor the interaction between parental gender and education (Wilks’ *Λ* = 0.993, *F*(12, 2709.541) = 0.569, *p* = 0.868, partial *η*^2^ = 0.002) was significant. These results indicate that parental education exerts an influence independent of gender.

One-way ANOVAs were conducted to explore the effect of father’s education level (1 = primary to 5 = university) on each outcome variable. Descriptive statistics showed similar means across education levels for legal cognition (*M*₁ = 4.60, *SD =* 0.70; *M*₂ = 4.62, *SD* = 0.62; *M*₃ = 4.55, *SD* = 0.68; *M*₄ = 4.63, *SD* = 0.68; *M*₅ = 4.49, *SD* = 0.80), natural environment satisfaction (*M*₁ = 3.41, *SD* = 0.70; *M*₂ *=* 3.50, *SD* = 0.74; *M*₃ = 3.61, *SD* = 0.49; *M*₄ = 3.55, *SD* = 0.64*; M*₅ = 3.37, *SD* = 0.80), and aggressive behavior (*M*₁ = 2.15, *SD* = 0.72; *M*₂ *=* 2.11, *SD* = 0.65; *M*₃ *=* 2.37, *SD* = 0.76; *M*₄ = 2.13, *SD* = 0.70; *M*₅ = 2.01, *SD* = 0.87). Homogeneity of variance was confirmed for all variables (all *p* > 0.05). The ANOVAs revealed no significant main effects of father’s education on legal cognition (*F*(4, 513) = 0.260, *p* = 0.903, *η*^2^ = 0.002), natural environment satisfaction (*F*(4, 513) = 1.382, *p* = 0.239, *η*^2^ = 0.011), or aggressive behavior (*F*(4, 513) = 1.323, *p* = 0.260, *η*^2^ = 0.010). Therefore, no post-hoc tests were performed.

In contrast, one-way ANOVAs on mother’s education level showed a different pattern. While mean scores were comparable across education levels for legal cognition (*M*₁ = 4.62, *SD* = 0.68; *M*₂ = 4.57, *SD* = 0.71; *M*₃ = 4.62, *SD* = 0.61; *M*₄ = 4.61, *SD* = *0*.63; *M*₅ = 4.53, *SD* = 0.68) and natural environment satisfaction (*M*₁ = 3.43, *SD* = 0.70*; M*₂ = 3.50, *SD* = 0.72; *M*₃ = 3.53, *SD* = 0.51; *M*₄ = 3.54, *SD* = *0*.70; *M*₅ *=* 3.64, *SD* = 0.62), greater variability was observed in aggressive behavior (*M*₁ = 2.13, *SD* = 0.71; *M*₂ = 2.02, *SD* = 0.65; *M*₃ = 2.53, *SD* = 0.73; *M*₄ = 2.21, *SD* = 0.69; *M*₅ = 2.25, *SD* = 0.87). Homogeneity assumptions were met (all *p* > 0.05). A significant main effect of mother’s education was found for aggressive behavior (*F*(4, 513) = 5.038, *p* = 0.001, *η*^2^ = 0.038), but not for legal cognition (*F*(4,513) = 0.158, *p* = 0.959, *η*^2^ = 0.001) or natural environment satisfaction (*F*(4, 513) = 0.736, *p* = 0.568, *η*^2^ = 0.006).

Tukey HSD *post hoc* analysis indicated that children of high school-educated mothers (Level 3) demonstrated significantly higher aggression (*M* = 2.53) than those with elementary (Level 1, *M*_diff = 0.398, *p* = 0.003) or junior high school-educated mothers (Level 2, *M*_diff = 0.513, *p* < 0.001). No other significant differences were observed among other educational levels. Results suggest maternal high school education is specifically associated with elevated aggression in offspring compared to lower educational attainment.

In summary, gender differences were negligible across all variables. Parental education showed a significant multivariate effect, driven primarily by mother’s education level on aggressive behavior, with children of high-school-educated mothers exhibiting the highest levels of aggression.

### 4.4 Moderating effect of natural environment satisfaction

The moderating effect of natural environment satisfaction was tested using the PROCESS macro (Model 1) (all variables were standardized before analysis) (Bolin, 2014). As shown in Table 2, after controlling for gender, age, and parental education levels, legal cognition significantly negatively predicted aggressive behavior. Additionally, the interaction term between natural environment satisfaction and legal cognition had a significant negative predictive effect on aggressive behavior, indicating that natural environment satisfaction moderates the relationship between legal cognition and aggressive behavior.

**Table 2:** The moderating role of satisfaction with the natural environment in the relationship between legal cognition and aggressive behavior.

Variables	Aggressive behavior			
	*β*	*t*	*p*	*CI*
Constant	−0.03	−0.12	0.903	[−0.56, 0.50]
Gender	0.07	0.84	0.399	[−0.10, 0.25]
Age	−0.001	−0.11	0.913	[−0.02, 0.02]
Father’s education level	−0.09	−2.00	0.045	[−0.19, −0.00]
Mother’s education level	0.14	2.70	0.007	[0.04, 0.24]
Legal cognition	−0.27	−5.22	<0.001	[−0.37, −0.17]
Natural environment satisfaction	−0.05	−1.24	0.214	[−0.15, 0.03]
Legal cognition * natural environment satisfaction	−0.11	−3.37	<0.001	[−0.17, −0.05]
*R* ^2^	0.082			
*F*	6.542		<0.001	

*N* = 518. Standardized regression coefficients with 95% confidence intervals (CI) are reported.

**Figure 1:**
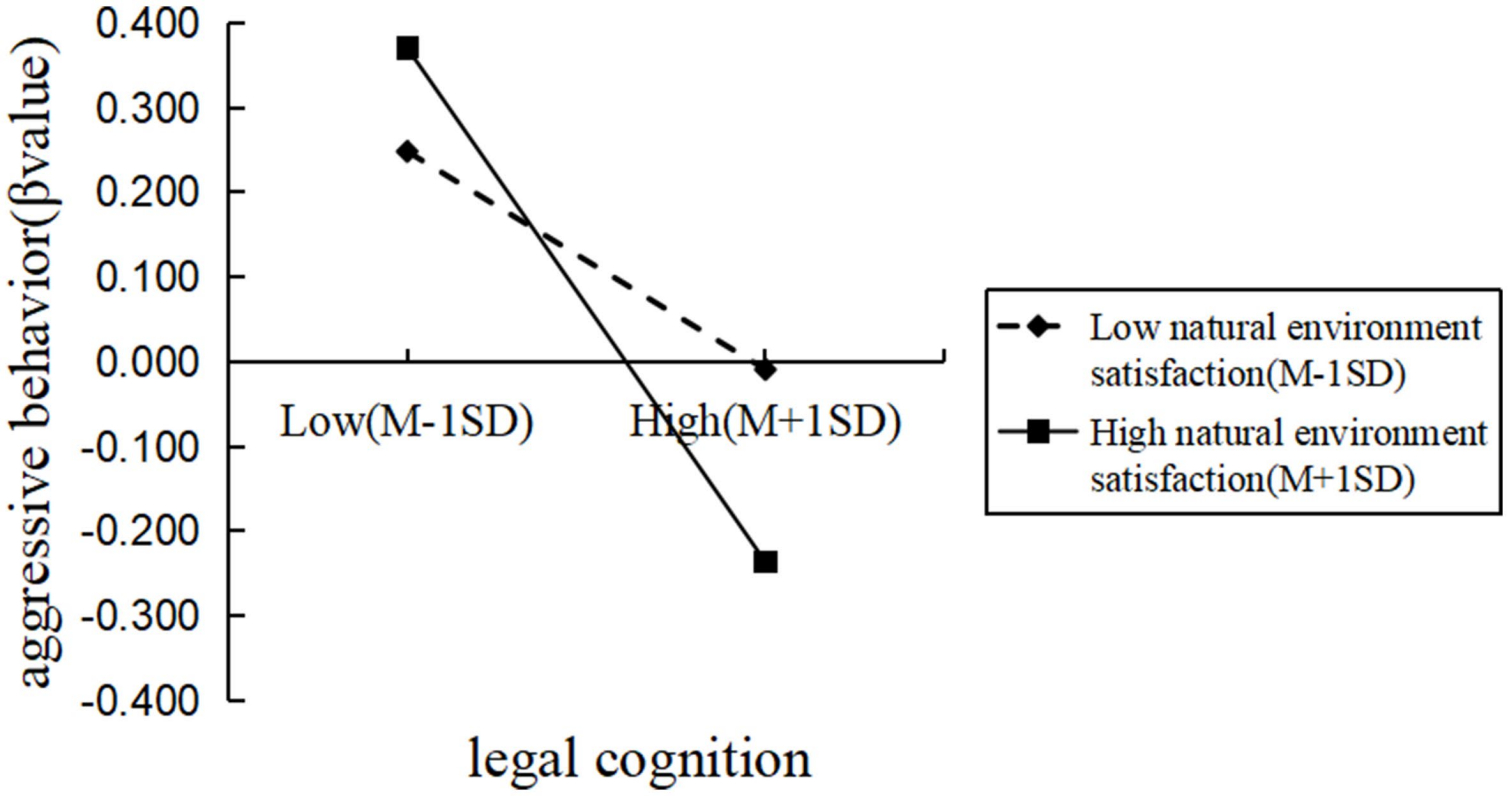
The moderating effect of satisfaction with the natural environment on the relationship between legal cognition and aggressive behavior.

To further examine the moderating effect of natural environment satisfaction, participants were divided into high and low natural environment satisfaction groups based on ±1 standard deviation from the mean. A simple slope analysis revealed that in the low natural environment satisfaction group, legal cognition significantly negatively predicted aggressive behavior (*β*simple = −0.16, *p* < 0.01). In the high natural environment satisfaction group, legal cognition also significantly negatively predicted aggressive behavior, but the predictive effect was stronger (*β*simple = −0.38, *p* < 0.001). These findings indicate that as individuals’ satisfaction with the natural environment increases, the negative predictive effect of legal cognition on aggressive behavior also strengthens. See Figure 1 for illustration.

## 5 Discussion

Our findings showed nonsignificant gender differences in aggressive behavior, legal cognition, and natural environment satisfaction, contrasting with Social Role Theory, which predicts observable differences due to gender-role socialization during adolescence (Eagly and Wood, 2016). Empirical evidence typically shows higher male aggression (Chiebuka et al., 2022), but our female-overrepresented sample (66.6%) did not replicate this pattern, suggesting either a weakening influence of traditional gender roles or methodological effects of sample imbalance. Notably, the attenuated gender differences in legal cognition and environmental satisfaction may indicate increasing gender neutrality in these domains. Additionally, while family SES, often indicated by parental education, usually affects child outcomes (Davis-Kean, 2005), we found that lower maternal education was associated with lower aggression, possibly reflecting stricter academic expectations from highly educated mothers in the Chinese context.

Consistent with previous research (Nivette et al., 2019), this study found that legal cognition negatively predicts aggressive behavior, indicating that individuals with higher levels of legal cognition are less likely to engage in aggressive behavior. Thus, H1 was supported. Existing research suggests that compared to juvenile offenders without significant aggressive behavior, those with aggressive tendencies are more likely to hold negative self-perceptions and worldviews, exhibiting cognitive distortions and negative attribution styles, such as overgeneralization and catastrophizing (Frey, 1999). Cognitive distortions have been widely recognized as a core issue in the study and treatment of aggressive and criminal behavior, with significant effect sizes observed in the relationship between cognitive distortions and externalizing behaviors (Helmond et al., 2015). Further research indicates that impulsive implicit cognitive processing in aggressive individuals is associated with increased trait aggression (Ireland and Adams, 2015). Moreover, enhancing cognitive control ability has been found to effectively reduce anger and aggression levels (Wilkowski et al., 2010).

Legal cognition not only reflects an individual’s rational understanding of the social control system, including their perception of the judicial system and legal authorities, but also embodies a positive evaluation of interpersonal and societal relationships. Individuals with higher levels of legal cognition tend to exhibit more positive self-perceptions and more rational social cognition. In the process of social information processing, they are more likely to interpret social cues from a perspective of fairness and justice and to resolve conflicts through legal and appropriate means (Tyler and Trinkner, 2018). In contrast, individuals with lower levels of legal cognition are more prone to developing negative attribution styles, perceiving the social environment as hostile and unjust, which increases the likelihood of aggressive reactions (Nivette et al., 2019). Therefore, legal cognition negatively predicts aggressive behavior, further emphasizing that legal education for adolescents should not only focus on imparting legal knowledge but also on fostering legal recognition and trust. By promoting a more positive social cognition model, legal education can help reduce aggressive behavior among adolescents.

Furthermore, this study found that natural environment satisfaction moderates the relationship between legal cognition and aggressive behavior. The results supported H2. Existing research has primarily focused on the influence of social environments on aggressive behavior, such as the effects of classroom environments (Werthamer-Larsson et al., 1991), the moderating role of maternal warmth in the relationship between extraversion and aggression in young childrenand the influence of teacher-student relationships on aggression in elementary school students (Liu et al., 2020), moderated by parent–child bonding (Yu et al., 2025). Additionally, studies have found a connection between urban green spaces and violent crime rates, with greater exposure to greenery associated with lower rates of violent crime (Kondo et al., 2017; LoSel and Farrington, 2012). These findings collectively suggest that environmental factors play a crucial role in shaping individual aggressive behavior.

This study further found that when individuals have higher levels of natural environment satisfaction, the negative predictive effect of legal cognition on aggressive behavior is stronger. In other words, individuals living in low-pollution, low-noise, and highly green environments are more likely to exhibit higher levels of legal cognition, which, in turn, helps regulate their cognitive processes and reduce aggressive behavior. These findings suggest that the natural environment influences not only emotional and stress levels but also cognitive regulation mechanisms, thereby enhancing the inhibitory effect of legal cognition on aggression. Individuals with high natural environment satisfaction are more likely to develop rational and clear behavioral cognition, leading them to handle conflicts in a more reasoned and lawful manner, thereby reducing aggressive behavior. This discovery highlights that the influence of legal cognition on aggression is moderated by environmental factors, suggesting that future research should further explore the role of environmental variables in the regulation of individual social behaviors.

The present findings indicated that the negative predictive effect of legal cognition on adolescents’ aggressive behavior had been stronger when satisfaction with the natural environment was high. According to the Protective Factor–Protective Factor (PFPF) Model, environmental satisfaction and legal cognition had jointly exerted a synergistic protective effect. From the perspective of social cognitive theory (Chen and Liu, 2019), legal cognition both shaped and had been shaped by environmental contexts, which in turn modulated adolescents’ behavioral responses. These results underscore the importance of integrating legal education with environmental improvements in interventions, promoting both effective socialization and psychological wellbeing among adolescents.

## 6 Limitations and future research directions

This study has several limitations. First, its cross-sectional design precludes causal inference; while lower legal cognition is associated with higher aggression, reverse or bidirectional effects are possible. Future research should use longitudinal or experimental designs to test causality. Second, convenience sampling and a gender-imbalanced sample may limit generalizability, warranting more representative sampling in future studies.

As the first study to examine natural environment satisfaction as a moderator between legal cognition and aggression, future research could employ behavioral experiments to further investigate this relationship and the robustness of the moderating effect. Interventions integrating legal education with environmental improvements, such as expanding urban green spaces, should be systematically tested, providing evidence to guide policymakers and educators in youth development and aggression prevention.

## 7 Conclusion

This study found that legal cognition significantly inhibits adolescent aggressive behavior, with natural environment satisfaction moderating this relationship. Adolescents with high legal cognition and high satisfaction with their natural environment exhibit lower levels of aggression, highlighting the combined importance of cognitive and environmental factors.

The findings emphasize the pivotal role of legal education: higher legal cognition promotes rational conflict resolution and constructive self-perceptions, reducing aggressive responses. Moreover, a well-maintained natural environment—featuring lower pollution, reduced noise, and greater green coverage—enhances the inhibitory effect of legal cognition on aggression.

Future interventions should integrate legal education and environmental improvements, fostering both cognitive understanding and favorable living conditions to support healthy adolescent development and provide empirical guidance for aggression prevention and social governance.

## Data Availability

The raw data supporting the conclusions of this article will be made available by the authors without undue reservation.
